# Side-by-side analysis of five clinically tested anti-EpCAM monoclonal antibodies

**DOI:** 10.1186/1475-2867-10-44

**Published:** 2010-11-02

**Authors:** Markus Münz, Alexander Murr, Majk Kvesic, Doris Rau, Susanne Mangold, Stefan Pflanz, John Lumsden, Jörg Volkland, Jan Fagerberg, Gert Riethmüller, Dominik Rüttinger, Peter Kufer, Patrick A Baeuerle, Tobias Raum

**Affiliations:** 1 Micromet AG, Staffelseestr. 2, 81477 Munich, Germany; 2 Micromet, Inc. 6707 Democracy Blvd., Bethesda, MD 20817, USA; 3 Institute for Immunology, Ludwig-Maximilians University, Goethestr. 31, 81333 Munich, Germany

## Abstract

**Background:**

Epithelial cell adhesion molecule (EpCAM) is frequently and highly expressed on human carcinomas. The emerging role of EpCAM as a signalling receptor and activator of the wnt pathway, and its expression on tumor-initiating cells, further add to its attractiveness as target for immunotherapy of cancer. Thus far, five conventional monoclonal IgG antibodies have been tested in cancer patients. These are murine IgG2a edrecolomab and its murine/human chimeric IgG1 antibody version, and humanized, human-engineered and fully human IgG1 antibodies 3622W94, ING-1, and adecatumumab (MT201), respectively. Here we compared all anti-EpCAM antibodies in an attempt to explain differences in clinical activity and safety.

**Methods:**

We recombinantly produced all antibodies but murine edrecolomab and investigated them for binding affinity, EpCAM epitope recognition, ADCC and CDC, and inhibition of breast cancer cell proliferation.

**Results:**

ING-1 and 3622W94 bound to EpCAM with much higher affinity than adecatumumab and edrecolomab. Edrecolomab, ING-1, and 3622W94 all recognized epitopes in the exon 2-encoded N-terminal domain of EpCAM, while adecatumumab recognized a more membrane proximal epitope encoded by exon 5. All antibodies induced lysis of EpCAM-expressing cancer cell lines by both ADCC and CDC with potencies that correlated with their binding affinities. The chimeric version of edrecolomab with a human Fcγ1 domain was much more potent in ADCC than the murine IgG2a version. Only adecatumumab showed a significant inhibition of MCF-7 breast cancer cell proliferation in the absence of complement and immune cells.

**Conclusion:**

A moderate binding affinity and recognition of a distinct domain of EpCAM may best explain why adecatumumab showed a larger therapeutic window in cancer patients than the two high-affinity IgG1 antibodies ING-1 and 3622W94, both of which caused acute pancreatitis.

## Introduction

Epithelial cell adhesion molecule EpCAM (CD326; 17-1A antigen) was among the first human tumor-associated antigens discovered [1]. It was initially identified by the monoclonal antibody (mAb) 17-1A after immunization of mice with human colorectal cancer cells [2]. Using a similar approach, the EpCAM antigen has been identified many more times and each time given the name of the respective monoclonal antibody [3,4]. For a long time, EpCAM was considered a mere cell surface protein mediating homotypic cell adhesion [5-7]. This function did not well explain its frequent and high expression in primary tumors and metastases [1,3], correlation of expression with poor survival prognosis [1,8], and its expression on tumor-initiating or cancer stem cells [9-12]. Only recently, EpCAM was shown to play a role in cell proliferation, signal transduction, and as a proto-oncogene [13-15]. EpCAM can undergo regulated intra-membrane proteolysis leading to release of its small intracellular domain EpICD [14]. In the cytoplasm, released EpICD combines with adaptor proteins FHL2 and β-catenin ultimately leading to formation of a large nuclear complex containing transcription factor LEF/TCF, which can turn on transcription of c-myc and cyclin genes and thereby drive cancer and stem cell proliferation [16-20].

EpCAM has been selected as target antigen for many immunotherapeutic approaches based on either antibodies or vaccines [1,21]. In 2009, an anti-EpCAM trispecific antibody called catumaxomab (Removab) obtained market authorization in Europe for treatment of malignant ascites in cancer patients [22]. Several other EpCAM-directed antibodies and antibody-based constructs are at various stages of clinical development [1,21,23]. The first monoclonal antibody ever tested in cancer patients was the EpCAM-specific murine IgG2a antibody 17-1A produced in ascites of mice [24,25]. This antibody was later produced by fermentation and called edrecolomab. Clinical evaluation of the antibody was mainly done in patients with colorectal cancer, either with metastatic disease [26] or in the adjuvant setting [27]. Objective responses were achieved only in a limited number of patients with metastatic disease [26]. However, a pivotal study in patients with surgically resected colorectal cancer Dukes' stage C randomized to observation or treatment with edrecolomab showed a significant clinical benefit, i.e., reduction of recurrence and death rate, and a benign safety profile [27,28]. Subsequent larger studies in Europe and the USA could not confirm edrecolomab's clinical activity in the adjuvant setting [29-31].

The high immunogenicity and short serum half life of murine antibody edrecolomab prompted the development of chimeric, humanized, human-engineered and fully human anti-EpCAM antibodies all sharing the human Fcγ1 portion. The human IgG1 isotype was selected for its superior potential of antibody-dependent cellular cytotoxicity (ADCC) and complement-dependent cytotoxicity (CDC) [32-34]. Only few patients have been treated with a human/murine chimeric version of edrecolomab, and then also in combination with GM-CSF to augment effector cell function and thereby potentially enhance the efficacy of the antibody [35,36]. The induction of an anti-idiotypic response was also explored [36] though no positive correlation between an anti-idiotype formation and clinical response to 17-1A antibody could be established in more than 60 colorectal cancer patients [37].

The human-engineered and humanized anti-EpCAM antibodies ING-1 and 3622W94, respectively, were developed and tested in clinical phase 1 studies in cancer patients [38-40]. Both had a relatively high binding affinity for EpCAM, had a maximum tolerated dose (MTD) of 1 mg/kg bodyweight, and their dose-limiting toxicity was acute pancreatitis [38,39,41]. Adecatumumab (MT201) is a fully human IgG1 antibody binding with intermediate affinity to EpCAM [42]. It has so far been administered to more than 240 patients with prostate and breast cancer in two phase 1 [43-45], and two phase 2 trials [45]. No MTD has been reached at 6 mg/kg when delivered as monotherapy [44,46], and up to 13 mg/kg were tolerated in a phase 1 study in combination with taxotere. Of note, no clinically manifest pancreatitis has been observed to date. Retrospective analyses indicated that a subgroup of patients with metastatic breast cancer having primary tumors expressing high levels of EpCAM showed clinical benefit by adecatumumab in terms of increased time to progression and reduced incidence of new lesions [45]. When combined with taxotere, a higher percentage of objective responses are observed with the antibody/taxotere combination than with chemotherapy alone [47].

Here, we have for the first time compared side-by-side edrecolomab in its murine and chimeric version, ING-1, 3622W94 and adecatumumab for their in-vitro biological characteristics, including binding affinity, epitope recognition, ADCC and CDC, and inhibition of cancer cell proliferation. Adecatumumab was unique in that it bound to an epitope encoded by exon 5 of EpCAM, and by inhibiting proliferation of MCF-7 breast cancer cells in the absence of effector cells and complement. Our data may help to better understand the differences in tolerability and signs of clinical activity of the five monoclonal antibodies.

## Methods

### Generation and purification of antibodies

Edrecolomab (murine anti-EpCAM; 17-1A; Panorex) was obtained as clinical trial material produced under GMP using a murine hybridoma cell line (Centocor Inc.). Human antibody adecatumumab (MT201) was generated by phage display-guided selection and produced by CHO cells as described previously [42,48]. The murine/human chimeric version of edrecolomab, and 3622W94 (hu323/A3) and ING-1 antibodies were generated by grafting according V_H _and V_L _DNA sequences onto a human IgG_1 _backbone. V_H _sequences were cloned into pEF-dhfr and V_L _sequences into pEF-ada expression vectors. These antibodies were expressed in CHO cells and purified by FPLC affinity purification using Protein G or A. Antibodies were eluted with 100 mM citrate buffer pH 2.3.

### Cell lines expressing murine, cynomolgus and human EpCAM and respective chimera

DHFR^- ^Chinese hamster ovary (CHO), Kato III, MCF-7, MCF10A cells and HEK293 cells were purchased from the American Type Cell Culture Collection (ATCC, Manassas, USA). CHO lines stably expressing human EpCAM (CHO-huEpCAM), murine EpCAM (CHO-mEpCAM), cynomolgus EpCAM (CHO-cynoEpCAM) and the chimera of cyno/human and murine/human EpCAM chimera were all generated by transfecting CHO cells with expression plasmids containing the respective cDNAs.

### Construction of cDNAs

In a first step, the cDNA encoding human EpCAM was modified by introduction of three silent recognition sites for restriction enzymes. The resulting construct allowed convenient exchange of defined human sequence fragments through their corresponding cyno equivalents in the context of the EpCAM full-length cDNA through a simple 'cut-and-paste' cloning approach. The cDNAs encoding different human/cyno EpCAM fragments were generated by gene synthesis. Chimeric constructs of verified DNA sequence were cloned into the pEF-dhfr expression vector to permit transient transfection and expression in HEK293 cells as well as stable expression in CHO cells.

Selection of clones and amplification of expression was performed in suspension with HyQ medium (HyClone, Logan, USA), supplemented with 20 nM methotrexate (Sigma-Aldrich, Steinheim, Germany). Transfected CHO cells were cultured in HyQ medium supplemented with 500 nM methotrexate at 37°C in a 5% CO_2 _chamber.

All *in vitro *assays were conducted in RPMI1640 medium (Biochrom AG, Berlin, Germany) supplemented with 10% fetal calf serum (Biochrom AG, Berlin, Germany), 100 u/ml penicillin and 100 μg/ml streptomycin (Biochrom AG, Berlin, Germany), 50 μM 2-mercaptoethanol (Invitrogen, Gaithersburg, MD), 1% non-essential amino acids (Biochrom AG, Berlin, Germany), 1 mM sodium pyruvate (Biochrom AG, Berlin, Germany), and 10 mM HEPES buffer (Biochrom AG, Berlin, Germany). Additionally, for MCF-7 cells 10 μg/ml insuline (sigma) was added and MCF10A cells were cultured as recommended by ATCC.

### ADCC assay

Kato III target cells grown under regular culture conditions were trypsinized for 5 min, sedimented by centrifugation and resuspended in culture medium to a concentration of 10^6 ^cells per ml. Cells (1 × 10^6^) were labelled by incubation with BATDA (Bis-(acetoxymethyl) 2,2':6',2''-terpyridine-6-6''-dicarboxylate), the hydrophobic esterified form of TDA, which diffuses readily through the cell membrane of viable cells and accumulates as membrane impermeable TDA inside the target cells due to hydrolyzation by intracellular esterases for 30 min at 37°C. After centrifugation at 350 g for 5 min cells were washed in medium 5 times. 5000 labelled cells/well resuspendend in medium were seeded into a 96-well plate as targets. Separately, peripheral blood mononuclear cells (PBMC) were prepared following conventional procedures (enriched by Ficoll-Hypaque gradient centrifugation), washed and resuspended at 6 × 10^6 ^per ml. Equal volumes of target and effector cell suspensions were mixed resulting in a final ratio of effector to target cells (E : T) of 50:1. This was followed by addition of an antibody solution, previously diluted in a 1:4 series, resulting in a final concentration ranging from 0.2 to 50,000 ng/ml. Cells were then incubated for 2.5 h at 37°C. After centrifugation at 150 g and 3 min, 20 μl of the supernatant were added to 100 μl Europium and incubated for 10 min for chelate formation. Quantification was performed subsequently by time-resolved fluorometry with a plate reader (WALLAC 1420 VICTOR^2^). The measured signal correlates directly with the amount of lysed cells. EC_50 _values are determined for each measurement using the four-parametric logistic regression model for the evaluation of sigmoidal dose-response curves. All experiments were performed in triplicate.

### CDC assay

KATO III cells grown under regular culture conditions were trypsinized for 5 min and resuspended in RPMI medium at a concentration of 10^6 ^cells per ml of media. Cells were labelled with BATDA as described in the ADCC section. To each 160 μl of cell suspension, 20 μl of cold human serum and 20 μl of the respective antibody dilution were added. Heat-inactivated human serum was used as control. After incubation of the cell cultures for 45 min at 25°C, cells were sedimented and analyzed as described above in the ADCC section. All experiments were performed in triplicates.

### Epitope mapping

For standard binding analysis, 1 × 10^5 ^to 2 × 10^5 ^cells were incubated with the respective antibody at concentrations ranging from 0 to 50 μg/ml in FACS buffer in a total volume of 50 μl for 30 min at 4°C. Thereafter, cells were washed twice in FACS buffer and incubated with FITC-labeled secondary antibody (Dianova Cat. No. DIA 920) for additional 30 min at 4°C. After two washing steps in FACS buffer, 10,000 to 20,000 events were analyzed.

### Cell proliferation assay

MCF-7 and MCF10A cells grown under regular growth conditions were trypsinized and seeded in the adequate medium at 3,000 cells/well in a 96-well flat bottomed plate. The following day, media was replaced with 100 μl media containing the respective antibodies at a concentration of 50 μg/ml. Cell proliferation was measured at the indicated time points using either the CyQUANT Cell Proliferation Assay Kit (Invitrogen) or Cell Proliferation Reagent WST-1 (Roche) according to the manufacturers' instructions.

### Determination of binding constants

Biacore analysis was performed using a Biacore 3000 reader (Applied Biosystems, Uppsala, Sweden). Soluble, recombinant extracellular domain of human EpCAM was produced and purified from the supernatant of stably transfected CHO cells [48]. EpCAM protein was coated to CM5 flow cells (Becton Dickinson) using the Amine Coupling Kit as described by the manufacturer. Binding studies were performed in a running buffer containing 10 mM HEPES, pH 7.4, 150 mM NaCl, 3.4 mM EDTA and 0.005% surfactant (P-20). KD and on/off rate constants were determined from sensorgrams collected with five different antibody concentrations.

## Results

### Binding Affinities

Binding affinities of the five recombinant antibodies was determined by surface plasmon resonance spectroscopy (BiaCore). Equilibrium dissociations constants (K_D _values) for binding to recombinant EpCAM coated on Biacore sensor chips were 1.5 and 2.1 μM for edrecolomab and chimeric edrecolomab, respectively, 100 nM for adecatumumab, and 190 and 160 pM for 3622W94 and ING-1, respectively (Table 1). K_D _values and on- and off-rate constants confirmed earlier data showing that 3622W94 and ING-1 were of higher affinity for EpCAM than adecatumumab and edrecolomab. On-rates of mAbs were rather similar, with adecatumumab showing the fastest on-rate. The two highest affinity mAbs, 3622W94 and ING-1 had very slow off-rates translating into a prolonged binding to the EpCAM target.

**Table 1:** Summary of characteristics of five clinically tested anti-EpCAM monoclonal antibodies analyzed side by side.

Antibody	Binding Affinity [nM]			Binding Domain	ADCC	CDC	Inhibition of MCF-7 Breast Cancer Cell Proliferation	Tolerability in Clinical Trials
	**K**_**D **_	**K**_**on **_**[M/s]**	**K**_**off**_**[1/s]**		**Mean EC**_**50 **_**in [ng/ml]**	**Mean at 20 μg/ml[%]**		
Edrecolomab	1530	2.81 × 10^4 ^	0.043	Exon 2	264	33	Not Significant	High

Chimeric Edrecolomab	2095	1.67 × 10^4 ^	0.035	Exon 2	671	60	Not Significant	High

3622W94	0.19	9.49 × 10^4 ^	1.8 × 10^-5^	Exon 2	38	70	Not Significant	Low (Pancreatitis)

ING-1	0.16	1.96 × 10^4 ^	3.2 × 10^-5^	Exon 2	14	63	Not Significant	Low (Pancreatitis)

Adecatumumab	91	3.46 × 10^5^	0.0316	Exon 5	175	29	Yes	High

### Binding Domains on EpCAM

**Figure 1:**
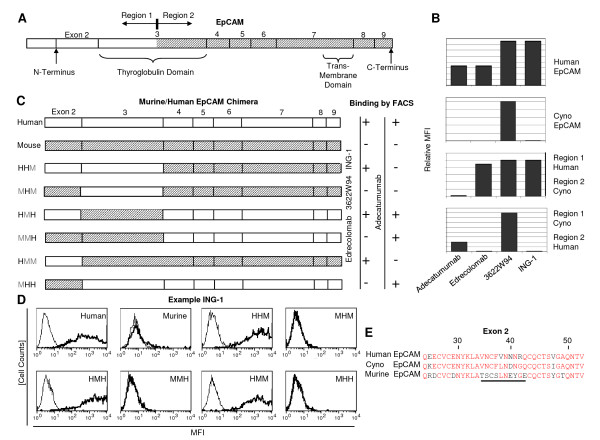
**Mapping of binding domains of four clinically tested anti-EpCAM monoclonal antibodies by use of CHO cells expressing cynomolgus monkey/human and murine/human chimera of EpCAM**. **a **Structure and exon boundaries of EpCAM. Open and shaded areas depict the subdomains used for making monkey/human chimera. **b **Binding of four clinically tested anti-EpCAM monoclonal antibodies to CHO cells expressing the indicated EpCAM proteins. **c **Structure of human/murine EpCAM chimera and results of FACS binding analysis (right side). Open areas show human sequences and shaded areas murine sequences. **d **Example of FACS binding analysis for ING-1 binding to transfected CHO cells. FACS histograms are shown with eight CHO cell transfectants. The bold line shows binding of detection antibodies in the presence of ING-1, the faint line binding in the absence of ING-1. MFI, mean fluorescence intensity. **e **Comparison of exon 2 sequences from human, cynomolgus and murine EpCAM. The line marks the sequence stretch of highest diversity.

A crude mapping of binding domains on EpCAM was done for the antibodies by using CHO cells expressing either human, cynomolgus monkey, or two human/monkey chimeric EpCAM proteins (Figure 1a). The two chimeric proteins exchanged human and monkey sequences in the middle of exon 3, which is encoding the thyroglobulin-like repeat of EpCAM. While all four antibodies bound to CHO cells expressing human EpCAM in FACS analysis (Figure 1b, top panel), the only antibody binding to all four CHO cell lines was 3622W94, indicating cross-reactivity with monkey EpCAM. All mAbs but adecatumumab bound to cells with a human N-terminal domain (third panel), while adecatumumab apparently recognized the C-terminal region 2 of human EpCAM (fourth panel).

A further mapping experiment used CHO cells expressing chimera between human and murine EpCAM (Figure 1c). Cells expressing murine EpCAM were no longer bound by mAb 3622W94. Edrecolomab, 3622W94 and ING-1 all showed robust binding in FACS to CHO cells expressing chimera that preserved the N-terminal sequence encoded by human exon 2 (i.e., HHM, HMH and HMM). As an example, binding of ING-1 in FACS scans to the various transfected CHO cells is shown in Figure 1D. Exon 2 encodes a small sequence around amino acid position 40, which is divergent between human, monkey and mouse EpCAM (Figure 1e). The underlined sequence does best explain the differences in species crossreactivity and may therefore represent part of the binding epitope for antibodies 3622W94, ING-1 and edrecolomab.

Binding of adecatumumab to a more C-terminal domain of human EpCAM (see Figure 1b) was supported by analysis using the murine/human chimeric EpCAM proteins. Only CHO cells expressing chimera containing sequences encoded by human EpCAM exons 4-9 were bound by adecatumumab (Figure 1c).

### Binding of Adecatumumab to an Exon 5-encoded Sequence of Human EpCAM

**Figure 2:**
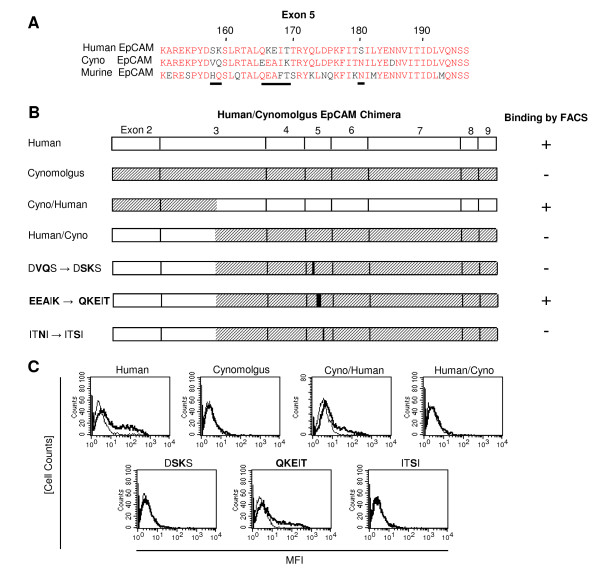
**Mapping of the binding domain of adecatumumab to exon 5 of human EpCAM**. **a **Comparison of exon 5 sequences from human, cynomolgus and murine EpCAM. The lines mark sequences of highest diversity between human and cynomolgus EpCAM. **b **Human/cynomolgus monkey chimera expressed on 293 cells for mapping the binding site for adeactumumab. The results of the FACS-based binding assay are shown on the right. **c **FACS histograms showing the binding of adecatumumab to 293 cells expressing the EpCAM constructs shown in b. The bold line shows binding of detection antibodies in the presence of adecatumumab, the faint line binding in the absence of the mAb. MFI, mean fluorescence intensity.

The binding site of adecatumumab in the C-terminal portion of EpCAM was further analyzed by HEK293 cells expressing chimera between human and cynomolgus EpCAM. Human and cynomolgus monkey EpCAM are highly conserved in this portion with major deviations limited to sequences in exon 5 around amino acid position 170 (Figure 2a). The deviating amino acids in the monkey EpCAM protein, which is not recognized by adecatumumab, were changed by directed mutagenesis to the amino acids of the human ortholog (Figure 2b). Exchange of amino acids EEAIK in the monkey EpCAM protein to QKEIT, as is present in the human EpCAM protein, restored binding of adecatumumab to HEK293 cells (Figure 2c). The binding signal by FACS to cells expressing the QKEIT variant of monkey EpCAM was as strong as the signal seen with cells expressing human EpCAM. This gain of binding identifies a small sequence in exon 5 as the binding site for adecatumumab on human EpCAM. Two other mutations, DVQS to DSKS and ITNI to ITSI, did not restore binding of adecatumumab to cynomolgus monkey EpCAM (Figures 2b, c).

### ADCC Activity

Target cell lysis by engagement of cytotoxic, Fcγ receptor-expressing immune cells (ADCC) or by fixation of complement (CDC) has been investigated for the five clinically tested anti-EpCAM antibodies. For ADCC, human peripheral blood mononuclear cells (PBMC) from five different healthy human donors were co-cultured with KATO III gastric carcinoma cells in the absence or presence of increasing concentrations of mAbs. Redirected lysis was monitored by release of a fluorescence dye from lysed cells.

**Figure 3:**
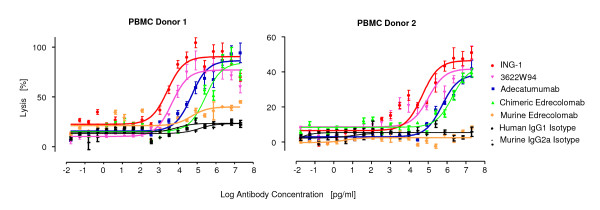
**ADCC activity of five clinically tested anti-EpCAM monoclonal antibodies**. ADCC was determined by dose response analysis as shown for 2 out of 5 human PBMC donors tested. KATO III gastric carcinoma cells were coincubated with human PBMC at an E:T ratio of 50:1 for 2.5 hours in the absence of presence of indicated mAb concentrations. Cell lysis was determined by TDA released from lysed cells chelated with Europium and quantified by the fluorescence of the Europium-TDA chelates formed. Results from triplicate determinations and standard deviations are shown.

A considerable donor variation for ADCC was observed as expected for non age-matched PBMC donors (Tab. 2). ING-1 followed by 3622W94 showed the highest potency of ADCC with a mean EC_50 _value for redirected lysis of 14 (0.76 nM) and 38 ng/ml (2.05 nM), respectively (Table 2). Examples for dose response curves obtained with two different donor PBMC are shown in Figure 3. Close to complete lysis of target cells was observed with PBMC from donor 1, and approximately 50% lysis with PBMC from donor 2. Adceatumumab was also relatively potent with a mean EC_50 _value for redirected lysis of 175 ng/ml (9.45 nM). While murine/human chimeric edrecolomab was rather potent with a mean EC_50 _value of 671 ng/ml (36 nM) and reaching a similar percentage of cell lysis as 3622W94, ING-1 and adecatumumab, the murine IgG2a version of edrecolomab was barely active with PBMC from donor 1, and not active with PBMC from donor 2 (Figure 3). Although murine edrecolomab induced very low levels of ADCC, its mean EC_50 _value was slightly lower than that of chimeric edrecolomab (Table 2).

**Table 2:** ADCC by five clinically tested anti-EpCAM monoclonal antibodies.

Antibody	Half-Maximum Lysis [ng/ml] Maximum Lysis [Percent]					**Mean Half-Maximum Lysis [ng/ml] ± S.D**.
	Donor					
	1	2	3	4	5	
**Edrecolomab**	49	n.d.	21	917	68	264 ± 436
	40%	n.d.	24%	37%	16%	

**Chimeric Edrecolomab**	277	1.910	69	515	596	671 ± 721
	84%	43%	19%	94%	44%	

**3622W94**	4.5	102	2.0	4.9	79	38 ± 48
	77%	42%	43%	82%	46%	

**ING-1**	2.6	48	0.9	8.2	10	14 ± 19
	90%	47%	23%	78%	54%	

**Adecatumumab**	50	537	11	100	176	175 ± 211
	86%	39%	27%	76%	53%	

### CDC Activity

For CDC, 10% human serum was added to KATO III gastric carcinoma cells and cell cultures incubated for 45 min. For inactivation of complement, a fraction of human serum was heat-treated and used as control condition, along with human IgG1 and murine IgG2a isotype control antibodies. CDC was monitored by fluorescent dye release from lysed cells.

**Figure 4:**
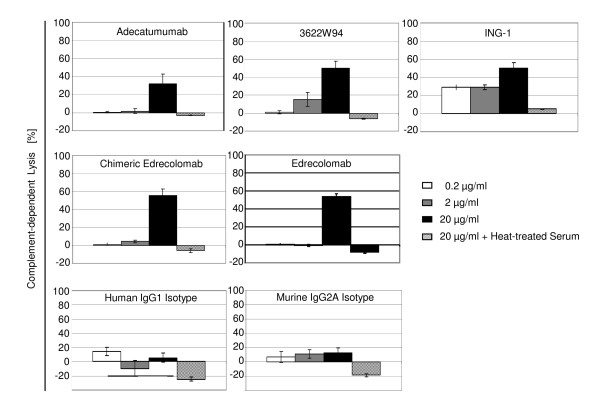
**CDC activity of five clinically tested anti-EpCAM monoclonal antibodies**. CDC was determined for three mAb concentrations. Human serum (10%) was added to cultures of KATO III gastric carcinoma cells and CDC after 45 min monitored by TDA released from lysed cells chelated with Europium and quantified by the fluorescence of the Europium-TDA chelates formed. Controls included incubation with heat-inactivated human serum, and human IgG1 and murine IgG2a isotype control antibodies. Results from triplicate determinations and standard deviations are shown.

All five anti-EpCAM mABs showed specific complement-mediated lysis of cancer cells in the range of 30-55% at an antibody concentration of 20 μg/ml (Figure 4). At 2 μg/ml mAb, only the two high affinity mAbs 3622W94 and ING-1 gave CDC signals. ING-1 was the only mAb active at 0.2 μg/ml. The two isotype control antibodies did not show significant CDC. In all cases, no CDC was observed with heat-treated human serum at the highest antibody concentration of 20 μg/ml.

### A Minute but Significant Impact of Effect on Breast Cancer Cell Proliferation

**Figure 5:**
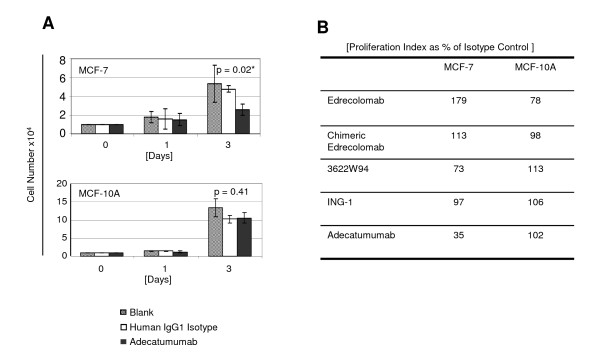
**The effect of adecatumumab on proliferation of breast cancer cell line and MCF-7 and normal breast epithelia line MCF10A**. The respective cell lines were seeded at 10^4 ^cells and cell numbers determined daily for three days. A human IgG1 isotype mAb and a blank served as controls. **a **Results from triplicate determinations and standard deviations are shown. P values between isotype control and adecatumumab values were determined by Student`s t-test. **b **The effect of five anti-EpCAM mAbs on proliferation indices of MCF-7 and MCF-10A cell lines as percent of isotype control antibody determined by CyQUANT cell proliferation assay. Results from a representative experiment are shown.

EpCAM was recently shown to have oncogenic potential and nuclear signalling activity in cancer cells [14], and to induce upon overexpression the proliferation of quiescent cells via c-myc [15]. Breast cancer cell line MCF-7 has been identified by knockdown experiments as being dependent on EpCAM for proliferation [13], which is why it was here selected for further characterization of antibodies. We tested the five mAbs at 50 μg/ml in cell culture for effects on the metabolism of breast cancer cell line MCF-7 and normal breast epithelial cell line MCF10A. Only adecatumumab showed a small but significant inhibitory effect on cell metabolism using a WST-1 assay (data not shown). It was the only mAb that showed after 3 days a significant 50% inhibition of MCF-7 cell proliferation when compared to a human IgG1 isotype control antibody (Figure 5a and 5b). Proliferation of normal breast epithelial cell line MCF10A was not significantly affected by any of the anti-EpCAM mAbs, including adecatumumab (Figure 5b).

## Discussion

Here we report the first side-by-side comparison of five clinically tested anti-EpCAM monoclonal antibodies. With the exception of adecatumumab and edrecolomab, mAbs were newly constructed by recombinant technology, produced by CHO cell clones and purified by a standard procedure. Their production in CHO cells may deviate from that in cell systems used for production of respective clinical test materials. As a consequence, certain mAbs investigated in the present study may differ in their carbohydrate composition from the clinically tested antibodies. Carbohydrates are known to influence ADCC and CDC activities of antibodies [49]. Hence results from ADCC and CDC assays have to be interpreted with caution. On the other hand, our standardized production of mAbs may allow for a better comparison of in-vitro properties than using clinical test samples, which are difficult to procure. N-linked carbohydrate structures as attached to the CH2 domain of IgG may be very similar among our four CHO-produced mAbs and, moreover, are not expected to impact binding affinity and specificity of the mAbs for the EpCAM antigen.

The most significant differences among edrecolomab, 3622W94, ING-1 and adecatumumab were found in their binding affinities and binding epitopes. Affinity constants derived from plasmon resonance analysis using immobilized, recombinant EpCAM protein matched well with values previously published for the mAbs. The binding epitopes of edrecolomab, 3622W94 and ING-1 are likely to be all contained in a short N-terminal sequence of EpCAM encoded by exon 2. In this sequence (see Figure 1e), position 42 is most divergent and therefore may best explain why cynomolgus EpCAM is not recognized by edrecolomab and ING-1, but by 3622W94, which may not depend on position 42 for binding. Future point mutation analyses are required to define the binding epitopes in greater detail. The binding epitope of adecatumumab was contained in a more membrane-proximal, exon 5-encoded sequence of human EpCAM (see Figure 2). Gain of antibody binding by replacing the short sequence EEAIK in cynomolgus EpCAM with the short human sequence QKEIT identifies the latter as part of the binding epitope for adecatumumab. Binding of adecatumumab to a different domain of EpCAM than recognized by the other mAbs apparently did not affect ADCC or CDC activity. It may, however, affect binding of the antibody to EpCAM on normal tissues, which may translate into a higher tolerability than reported for ING-1 and 3622W94. The much slower off-rates of ING-1 and 3622W94 compared to adecatumumab and edrecolomab may likewise determine the therapeutic window and anti-tumor activity. High-affinity antibodies were shown to poorly penetrate into tumor tissue while an affinity of 10^-7^-10^-8 ^M, as observed here for adecatumumab, was optimal for tumor penetration of anti-Her-2/neu single-chain antibodies [50].

All four human IgG1 mAbs showed robust ADCC activity leading to similar degrees of target cell lysis at the plateau of dose response curves. However, their EC_50 _values for redirected lysis were different and appeared to correlate with their respective binding affinities as determined by plasmon resonance spectroscopy. In addition, EC_50 _values showed considerable variation for two non age-matched PBMC donors. It has been reported that CD56-positive natural killer (NK) cells are the main effector population causing ADCC by anti-EpCAM antibody adecatumumab[51]. Elder donors were found to have higher amounts of NK cells but with lower activity compared to younger donors [52]. Here we paid attention to using the same donor PBMC for comparing all antibodies. Although the two PBMC donors gave different EC_50 _values for ADCC for each antibody the overall ranking of the antibodies' ADCC was conserved, suggesting that differences among the antibodies were not due to donor variation but to instrinsic properties. The murine version of edrecolomab performed poorest in ADCC with human effector cells. This may be due to a reduced compatibility of its murine Fcγ2a portion with Fcγ receptors on human immune effector cells. This notion has recently been demonstrated by comparing 'murinized' IgG2a and human IgG1 versions of adecatumumab [53].

At 20 μg/ml, all five mAbs showed robust and comparable levels of CDC during a 45-min assay. Only ING-1 and 3622W94 showed CDC at lower antibody concentrations, which again are in line with their higher binding affinity as seen in Biacore analyses. As reported earlier, adecatumumab and murine edrecolomab showed equal CDC [42], whereas other investigators could not detect any CDC for murine or chimeric edrecolomab [54]. Our data indicate that all but murine edrecolomab should be able to eliminate cancer cells in patients by both ADCC and CDC.

The findings that EpCAM is a proto-oncogene and signal transducer [9,14,15] raise the possibility that anti-EpCAM antibodies may be able to interfere with the proliferative signal transduction cascade initiated by EpCAM. Of all five mAbs tested, only adecatumumab could specifically and significantly reduce proliferation of breast cancer cell line MCF-7 but not of normal epithelial cell line MCF10A. EpCAM knockdown experiments in MCF-7 cells have shown that cells cease to proliferate, migrate and invade soft agar, indicating that this cancer cell line is dependent on EpCAM signalling [13]. Future studies will investigate whether the binding of adecatumumab to its membrane-proximal epitope interferes with proteolytic activation of EpCAM. At the same time, it can be studied whether murine edrecolomab is promoting the proteolytic activation of EpCAM leading to the higher proliferation index observed here for MCF-7 cells. It is tempting to speculate that the unique binding specificity of adecatumumab, its not overly high affinity, robust ADCC and CDC, and its inhibitory effect on cell proliferation may in their combination be important for producing signs of clinical activity and a good tolerability in more than 240 patients treated to date [43,45,46].

## Conclusions

From the present in-vitro data, the induction of acute pancreatitis by mAbs 3622W94 and ING-1 is best explained by their high binding affinities for EpCAM seen in Biacore analyses, in particular their very slow off-rates. High binding affinities may also best explain their superior CDC and ADCC activities at low concentrations. With a high affinity and slow off-rate, such antibodies could very well reach a surface density on cells of normal tissue, which is sufficient to trigger ADCC and CDC reactions even if low numbers of EpCAM molecules are accessible. Should EpCAM on normal tissue be mostly in complex with protein partners, e.g., with claudin-7, tetraspanins or CD44, high affinity antibodies may compete for their binding to EpCAM and permanently displace EpCAM-associated proteins while antibodies with lower affinity should be inefficient to do so.

## Competing interests

MM, AM, MK, DRa, SM, SP, JL, JV, JF, GR, DRu, PK, PB and TR are employees of Micromet, Inc., and have equity positions in the company. Micromet is focused on the development of BiTE antibodies for the treatment of malignant diseases.

The present study has not been used for a new patent filing. Funding of this study has been supplied by Micromet Inc., a publically listed company.

## Authors' contributions

MM conceived the study, participated in its design and coordination, and wrote a first draft of the manuscript. AM performed most of the experiments. MK carried out the biacore analysis. DRa, SM, SP, JL, JV participated in the molecular cloning of antibodies and FACS analyses. JF, GR and DRu provided critical input to the manuscript. PK and TR participated in the coordination of the study and participated in its design. PB conceived the study together with MM, and finalized the mansucript. All authors read and approved the final manuscript.
